# Characterization of grazing behaviour microstructure using point-of-view cameras

**DOI:** 10.1371/journal.pone.0265037

**Published:** 2022-03-18

**Authors:** Elvira Sales-Baptista, Maria Isabel Ferraz-de-Oliveira, Marina Terra-Braga, José António Lopes de Castro, João Serrano, Manuel Cancela d’Abreu

**Affiliations:** 1 Departamento de Zootecnia, Mediterranean Institute for Agriculture, Environment and Development (MED), Universidade de Évora, Évora, Portugal; 2 Master 1 Biologie-Agronomie-Santé, Parcours Comportement Animal et Humain Université de Rennes, Rennes, France; 3 Departamento de Engenharia, Mediterranean Institute for Agriculture, Environment and Development (MED), Universidade de Évora, Évora, Portugal; Universidad de Costa Rica, COSTA RICA

## Abstract

Grazing patterns, intake structure, and diet selection are dynamic responses to animals’ feeding environment. This study uses video sequences from animal-borne cameras to capture time- and scale-dependent grazing behaviour variables related to sward explanatory conditions. We observed grazing ‘through’ the sheep’s eyes using point-of-view (POV) cameras coupled with event logging software. Time-specific sward features were measured by sampling ‘really’ grazed patches identified by applying a global navigation satellite system (GNSS) precision-grazing approach. Sward variables on a Mediterranean native sward were measured for two years during the active spring plant-growth cycle. Overall, the results demonstrate that POV cameras were able to capture grazing behaviour fine-tuning to changes in sward characteristics. Sheep compensate for the decrease in sward quantity and nutritive value by increasing the size and duration at each behavioural scale (i.e., meal, bout, and station) while increasing the bout rate and decreasing the station rate. Diet composition also changed as sward matured. The proportion of forbs in the diet remained high in early and late spring, and forbs and legumes were preferred to grasses in early spring. Grazing selectivity was more pronounced in late spring, with sheep favouring the middle stratum of the sward’s vertical structure, preferring green vegetative material, while enlarging the feeding niches’ span and spending more time at each niche, consequently reducing the station rate. Although data collected by individual animal-borne POV cameras were representative of the flock behaviour, they may underestimate the total grazing time outside major meals. The results indicate that the use of animal-borne video cameras is suitable for assessing variations in sheep grazing behaviour patterns in complex swards.

## Introduction

Feeding is a key behaviour that affects the amount, type, and rhythm of nutrients ingested [1], thus having a significant impact on production efficiency, which, in turn, is linked to economic and environmental consequences. Therefore, improving feed efficiency is a major husbandry goal.

In general terms, more efficient animals spend less time eating and eat faster, which has been reported for cattle [2, 3], sheep [4] and pigs [5]. While the development of electronic identification coupled with automatic feeders has allowed accurate measurements of feeding behaviour components (e.g., frequency and duration), understanding the feeding behaviour of grazing livestock remains a challenge. Moreover, characterizing the components of feeding behaviour is essential to gain an understanding of what differentiates individuals and the feeding circumstances in which they excel. Several methods can be used to study the amount of feed ingested and the dietary choices (recently reviewed by [6]); however, most methods (e.g., oesophageal fistulas, faecal n-alkanes, and faecal near infrared spectroscopy [NIR]) are unable to determine the temporal and spatial dimensions of the feeding behaviour, which can only be obtained when studying where animals are, what they are doing, and when they are doing it [7, 8]. Direct visual observation of animal behaviour during the day is relatively straightforward but can be time-consuming, uncomfortable, and tedious. Additional drawbacks of direct observation are darkness and the distance between observer and animal. Both limit the range of vision, the first hampering observations of nocturnal activities [9] and the second precluding sight of details, such as occurrences at the oral behaviour scale [10]. When the focus of observation is the interface between animal and sward, detailed information on the structure of bites by direct visual observation requires a trained observer-animal pair moving in proximity (0.5 m–1 m) [11]. In such conditions, a bite-coding grid is used, which is based on plant features such as the shape and size of the selected parts and how they are harvested [11–14]. In structurally complex swards (biodiverse plant mixtures with contrasting morphology and anatomy), a bite-coding grid with more than 40 bite types would be needed, making it impractical. Additionally, it is difficult to predict how the presence of an observer may alter the behaviour of the grazing animal. The use of sensors such as electromyography, accelerometers, microphones, pressure sensors, and jaw switches (reviewed by [15]) may avoid the observer effect. However, despite conveying information on grazing patterns, sensors cannot provide information on forage selection. The use of handheld or automatic video cameras may overcome some of the drawbacks of direct visual observation and provide a complete record of behavioural activities that can be analysed later in lab conditions. Automatic video recordings have previously been used in experiments on feeding preferences at the bite scale both indoors [16, 17] and outdoors (on pasture) [18].

Within the scope of video recording, a step forward is the use of animal-borne cameras to assess grazing behaviour. One primary benefit is minimal disruption to the individual and surrounding environment. The methodology has the merit of capturing images from the animal’s point-of-view, revealing information on activity patterns, foraging strategies, and diet selection. Animal-borne cameras have been used in ecology studies for more than three decades. To our knowledge, POV cameras have not been used before on sheep, although they have been used on feral and domestic cats [19, 20], and on wild species (recently reviewed by [21]).

We hypothesize that POV cameras provide video sequences that can be used as a new method to capture feeding behavioural data on free range grazing sheep together with the spatiotemporal hierarchy framework of grazing behaviour, which is described briefly in the next section.

Considering the characteristic seasonal changes in sward height and the botanical composition of Mediterranean pastures, our aim was to ascertain whether POV cameras could be used to determine the microstructure of intake (the study of kinetics of food intake during feeding episodes in varying feed conditions), particularly (i) the variation in frequency and duration of meals, feeding bouts, and feeding stations and (ii) the variation in diet selection. For definitions of microstructure and related terms, see the glossary in the supporting information (S1 Table).

## Components of grazing behaviour and foraging dynamics

**Fig 1:**
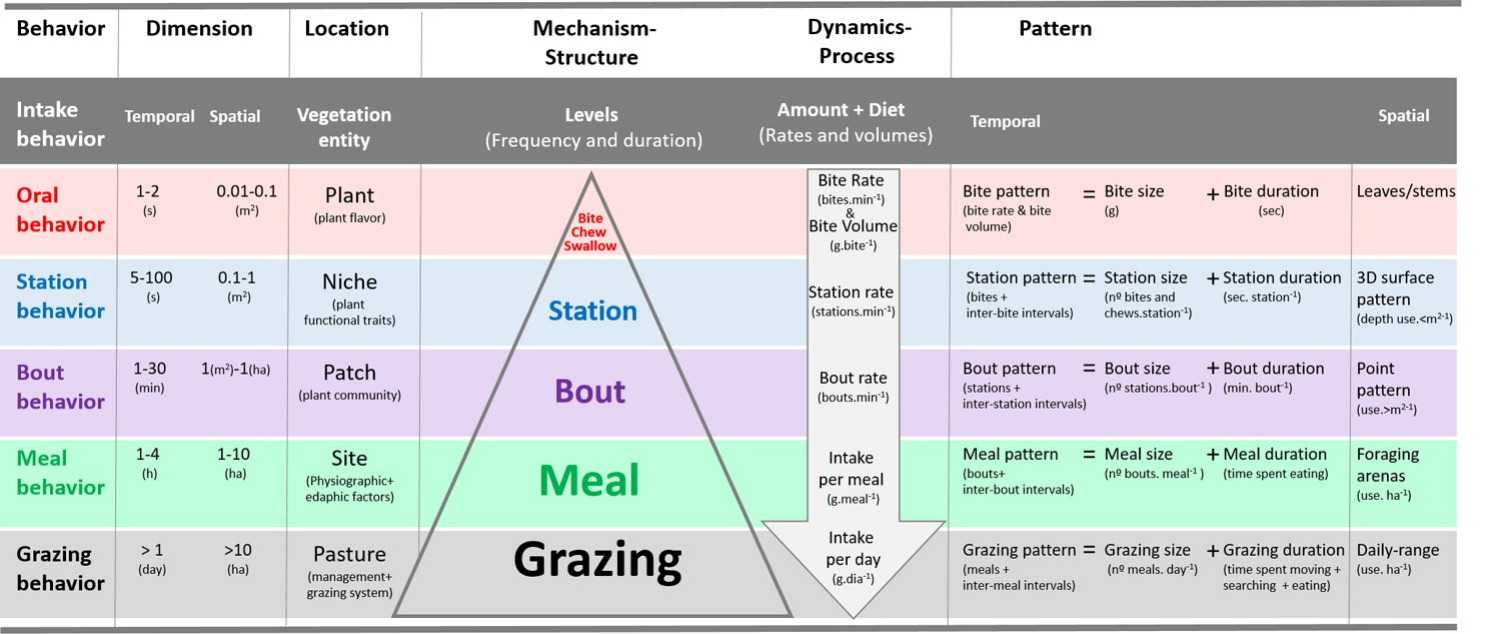
Components of grazing behaviour. Rows represent scale levels (behaviour units), columns represent hierarchical perspectives (dimensions, locations, structures, dynamics, and patterns). Patterns, at each scale, are generated by the distribution of frequencies, durations, rates, and volumes (based on the original conceptual model of ecological hierarchy of [24] and on contributions of [7, 15, 25]).

Grazing behaviour is organized in a hierarchical, nested manner where activities are behavioural units displayed as levels of a pyramid (Fig 1; for a comprehensive review, see [7]). The bottom level of the pyramid is grazing, a series of foraging actions, such as searching (wandering, exploring, selecting) and consuming (grabbing, biting, chewing, swallowing). Grazing is discontinuous due to other daily activities such as rumination or rest. Within grazing, consumption actions are thus divided into several meals, the next level of the pyramid. A meal starts when the animal begins to ingest and ends when the animal makes no further movement to graze for at least an hour. Within a meal, the length of time that an animal eats without stopping is a bout. Bouts are foraging sequences, where animals continuously eat with their heads down, although they may be walking slowly. A bout ends whenever the animal interrupts the sequence by raising its head, (e.g. walking to another location). Within a bout, whenever an animal is grazing without moving its front feet, we have a grazing station, which in turn is characterized by a cluster of bites. Each activity occurs in different spatial and temporal dimensions that are fundamentally interlinked. For instance, a meal is observed as a peak of eating activity occurring at a specific site (spatial dimension) and with a specific duration (1–4 h, temporal dimension) (S1 Table).

Each level embodies a behavioural unit of the grazing behaviour with its own spatiotemporal location, structure, dynamic, and pattern. This hierarchical organization can be adapted to quantitative and qualitative variations in the environment since grazing animals, at each behaviour scale, may alter the dynamics, modifying both total intake and diet selection [22–25]. Intake changes are linked to variations in both length of grazing time and number of events, defining a structure. Intake variations are also connected to the time period of occurrence, with time-budgets allocated to behavioural activities defining patterns. For instance, the grazing pattern results from the distribution of time spent in grazing disconnected by intervals of other behavioural activities within a time period (e.g. day) and may shift when grazing animals change the major periods of grazing activity throughout the day [23, 26].

Diet changes result from animal preferences as well as opportunities and are primarily connected to the spatial dimension. The flexibility of grazing behaviour results from tuning the duration and frequency at the different behaviour scales according to vegetation characteristics, which ultimately depends on animal decisions.

To discriminate the components of a meal in its finer temporal pattern, we adapted the schematic microstructure of intake proposed by [27] for rodents to ruminants. This approach has the merit of separating orosensory stimulation from gastrointestinal control [28, 29]. The oral behaviour links the hedonic and sensory animal response (taste, smell, sight) to chemical properties (nutrients, secondary metabolites) [16] and functional traits (e.g. flowering/heading time, leaf/stem ratio, plant height) of the selected plant.

**Fig 2:**
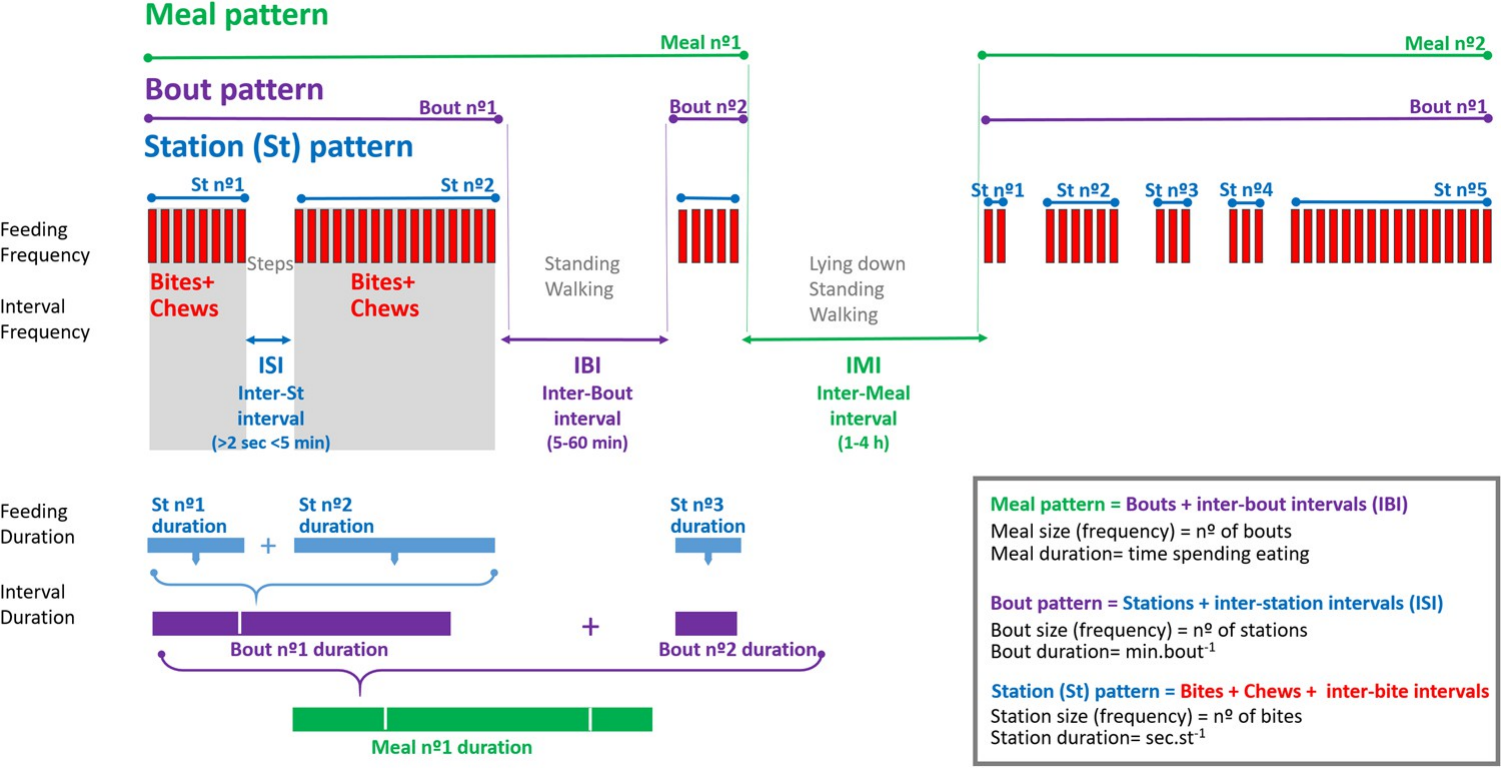
Components of a meal microstructure and definitions. (Adjusted for grazing ruminants, based on [28, 29]).

The structure of a meal consists of bite sequences divided by intervals [28, 29]. The number of bites occurring during a station affects station size and duration (c.f. Fig 2). The sum of stations and intervals between (steps while moving with the head down) defines a grazing bout. Ultimately, the size of a meal depends on the number of bouts separated by other behavioural activities, such as standing or walking. The size and duration of a meal define the meal pattern, and the meal with the longest duration is the major meal [12, 26]. The same rationale and organization are used for each behavioural unit of the grazing behaviour.

Analysing meal microstructure is a way of asking animals how they perceive and adapt to their feed resources. Thus, by quantifying changes in meals, bouts, stations and bites we are able to reveal relations usually hidden. Such findings will potentially lead to a better understanding of the performance of grazing animals and will have implications in the design of grazing strategies.

## Materials and methods

In this study, two experiments were carried out using POV cameras to measure grazing responses. In the first, we explored the potential ability of video sequences to uncover the relationship between the grazing behaviour of sheep and feed resources. In the second, we tested the validity of the method with direct observation of group behaviour. Additionally, we investigated the overnight use of POV cameras to explore nocturnal grazing behaviour.

### Experimental site

The field research took place at the University of Évora’s Mitra Experimental Station, Portugal, (38°31’52"N, 8°00’33"W) in 2016 and 2017. The mean elevation of the study site was 280 m, and the predominant soil was a Cambisol derived from granite. The site has a hot summer Mediterranean climate, with the regional average temperature ranging from 5.8°C in January to 30.2°C in July and August, and annual rainfall of 600 mm, 72% of which occurs between October and March.

The experimental site was a 2.3 ha silvopasture paddock, with holm oak trees (*Quercus rotundifolia*

[Lam.]) covering 15.7% of the area (tree density of 9.1 ha^−1^), and a native pasture where the frequency of occurrence of forbs, grasses, and legumes was 52.1%, 28.5%, and 19.4%, respectively. A mobile custom-made sheep-handling system was available in the paddock.

### Animals

**Fig 3:**
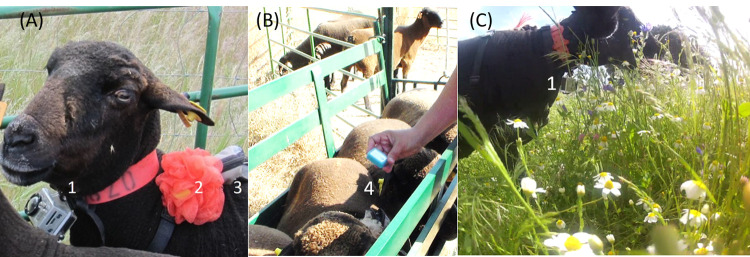
Behavioural sensors setup. (A) Sensors fitted on a sheep; (B) ewes in sheep–handling system while placing the global navigation satellite system (GNSS) in the pouch; (C) ewe grazing wearing the sensors, captured by another ewe. (1) POV camera (GoPro^®^) attached to collar; (2) ewe identification mark attached to harness; (3) GNSS receiver pouch attached to harness; (4) GNSS receiver (Mr. Lee CatTrack^®^).

Fifteen six-year-old, non-lactating, non-pregnant Black Merino ewes (69.8 kg ± 1.7 kg live weight), were stocked continuously during the experiment. The ewes were originally part of a flock of 60 ewes managed in a year-round extensive grazing system. Five ewes (experiment 1) and three ewes (experiment 2) were selected to carry behavioural sensors (Fig 3) for collecting data on grazing behaviour and location (c.f. Behavioural Sensors section). The ewes were fitted with collars, harnesses, and a colourful mesh pouf for ease of individual visual identification. The same animals were used in each experiment, and POV cameras were not mounted on different animals.

### Trial layout and experimental design

Two experiments were designed to evaluate the use of POV cameras to measure grazing response. Animals were the experimental unit, and we used repeated-measures designs involving multiple measurements on each experimental unit that functions as an experimental block. Experiment 1 covered the spring pasture growth, assuming that more pronounced behavioural adaptations occur in this period. Experiment 2 aimed to determine whether behaviour variables captured with POV cameras could represent the behaviour of the entire flock.

Behavioural data were collected in both experiments using POV cameras (screenshots shown in Fig 9) and GNSS receivers (c.f. Behavioural Sensors section). The ethogram of observed behaviours is presented as supporting information (S2 Table). In experiment 1, recording periods were set to cover morning and afternoon meals, whereas in experiment 2, an entire day was targeted. Before each recording period, the flock was gathered to replace POV camera batteries. The GNSS receivers were replaced weekly.

Experiment 1 was carried out from 4 April to 13 June 2016 across 11 consecutive weeks using a flock of 15 ewes. The whole flock was used for behavioural observations, and 5 of the flock of 15 animals were used for video recording. Video recording was restricted to daylight hours. Recordings from the same 5 animals were conducted once a week and used for extracting behavioural data, totalling 110 observations (5 animals × 11 weeks × 2 meals). Because of the environmental differences occurring between weeks, data were clustered in two seasons (early and late spring) of 5 weeks each, corresponding to the sward vegetative and reproductive stage, with a 1-week interval between seasons. Time periods considered cover morning (9am–12pm) and afternoon (6–9pm) to capture expected peaks of grazing activity, resulting in a daily total recording time of 6–8 hr of video sequences per ewe. Positions were recorded by GNSS loggers.

Experiment 2 was carried out from 19 April to 19 May 2017. The aim of the experiment was to determine whether the grazing behaviour captured by POV cameras could be used to represent the behaviour of the entire flock. The POV cameras were carried by 3 ewes in the flock of 15. The POV cameras were used to assess individual ewes’ behaviour through focal sampling (behaviour of an individual animal is recorded continuously). Video records were made for a 24 hr cycle (using an infrared POV camera for the nocturnal period) and were repeated three times (using the same three ewes) with a 2-week interval. Each video sequence matched a period of the day, totalling five time periods (6–9am, 10am–1pm, 2–5pm, 6–9pm, and 9pm–6am). Simultaneously, flock behavioural activity (S2 Table) was measured from 6am to 9pm by direct observation using 10 min intervals with instantaneous scan sampling [30] (the behaviour of all individuals in a group of animals is recorded, each animal in turn, instantaneously). For later comparison of both datasets, data were analysed for each hour and again clustered in five periods of 170 minutes each. A repeated-measures crossover design was used to reduce error variance associated with inter-individual differences and to increase the statistical power. Video recording matched the moon quarters (third quarter on 19 April and 19 May; first quarter on 3 May) to have similar nocturnal light conditions.

### Pasture measures

The study area was virtually divided into 60 regular patches of 380 m^2^ each. Sheep are known to adjust their behaviour to maintain group cohesion whenever the space allowance for each animal is over 200 m^2^ [31]. The dimension of the defined patches (380 m^2^) allows for a maximum dispersal distance within a group (social distance) to ensure the location of the entire flock within one patch. Each patch represents a resource unit. Before forage sampling, each patch was assigned to one of the two sets (visited vs not-visited by ewes) by GNSS-tracked sheep movements. Data on animals’ georeferenced locations were downloaded each week. The last 72 hours of each week were analysed using geospatial software (c.f. Behavioural Sensors section), and visited vs not-visited patches were newly defined. Within the visited patches, different behavioural activities could occur; therefore, we focused on grazing activity on the assumptions that peaks of grazing events occur at dawn and dusk, and longer time spent grazing on a patch is related to greater dietary preferences. The three most-grazed patches were chosen for vegetation sampling each week. Forage quantity and nutritive value in relation to seasonal changes were evaluated weekly.

For each most-grazed patch, sward was randomly sampled using three quadrats (0.5 m × 0.5 m). Herbage within each quadrat was cut 2 cm above ground level using battery grass shears (Florabest, Lidl Stiftung & Co. KG, Neckarsulm, Germany) and processed to estimate forage mass (dry matter [DM] kg.ha^−1^). Nutritive value was assessed by measuring DM (ISO 6496:1999, [32]), crude protein (CP; Dumas method, ISO 16634–1;2008, [33]), and neutral detergent fibre (NDF; ISO 16472:2006, [34]). Before cutting the forage, the average sward height was measured using a wooden folding rule. Herbage bulk density (mg.cm^−3^) was calculated by dividing forage mass by the corresponding average sward height.

A nadir georeferenced photo captured 0.8 m above ground with a GoPro^®^ camera mounted on a pole and centred on the sampling quadrat was also obtained weekly before collecting the forage sample. To estimate the percentage of ground cover by plant species on the photoplots, a uniformly spaced 8 × 8 digital grid (i.e., 64 intersections) was superimposed on the digital image area defined by the sampling quadrat. The number of ‘hits’ on the plant species within the intersections of the grid was registered, and quantitative occurrence of species was then calculated as the percentage of total intersections. Species percentages were later grouped and converted to functional group percentages [35].

Inside each patch, several feeding niches were available in proportions that changed across the plant growth cycle. Feeding niches are locations within plant communities of a patch and distinguished by their functional traits assessed by visual estimation of the vegetation eaten by the animals (using the video sequence from POV cameras). Functional traits are responsible for the species’ response to competition, environmental stress, and disturbance, within the plant community and across environmental gradients. Those core plant attributes are linked to plant species’ ecophysiology and include, for example, flowering/heading time and leaf area or plant height [36], affecting plant colour and architecture. The identification of a feeding niche is possible since when the animals stop to eat (stations; Fig 1), the sward targeted by its mouth is visible (Fig 9). For each week, we assessed feeding niches used by the ewes. We defined 12 feeding niches by combining three features: plant functional group (grasses, forbs, and legumes, inferred from the plant shape recognized in the video image), sward vertical profile (surface vs middle-strata level, inferred from the position of the animal’s chin in relation to plants’ vertical strata in the video images), and plant maturity (greenish vs yellowish [37], inferred from the plant colour evidenced in the video images). Although 12 potential feeding niches were defined, only 9 were observed in the video sequences. Mature legumes in both vertical strata were non-existent or undetected. Mature forbs, in the surface strata of the vegetation, were also undetected.

As behavioural components adjust to pasture conditions, we used the pasture condition index (PCi; [Eq 1]) to capture the relation between forage nutritive value and quantity as sward plants mature. The loss of sward nutritive value is denoted by the relation of fibre and protein, and we use the pasture quality degradation index (PQDi; [Eq 2] [38]) to reflect it. The quantitative sward attributes are indicated through the relation of forage mass and sward height.

PCi=PQDi/BD (1)

PQDi=NDF/CP (2)

where CP is pasture crude protein content (%), NDF is neutral detergent fibre (%), and BD is bulk density (mg.cm^−3^).

### Behavioural sensors

For records of daylight behaviour, GoPro® Hero2 video cameras (GoPro Inc., San Mateo, CA, USA) were attached to the ewe’s collar and set at a fixed angle to enable chin visibility (Fig 3). Battery life was extended (4 hr) using an extra battery (GoPro® battery backpack). The fully equipped camera weighed 244 g, which represents approximately 0.4% of the animal’s live weight. Five cameras were used in each experiment. In Experiment 1, the five cameras were randomly allocated to each of the five ewes weekly. In experiment 2, the five cameras were exchanged among the three ewes to cover the diurnal time periods of the experiments.

Additionally, in Experiment 2, we used one infrared night-vision PatrolEyes® camera (PatrolEyes, Ada Township, MI, USA) to capture nocturnal behaviour [9]. Extra battery life was provided using an off-the-shelf power bank, enabling 10 hr of filming. The fully equipped infrared camera weighed 400 g (0.6% of the average animal’s live weight).

For georeferencing sheep locations, GNSS receivers weighing 22 g (Mr. Lee CatTrack^®^, Catnip Technologies Limited, Anderson, SC, USA) were used. Positions were recorded at 5 min intervals, and GNSS receivers were replaced weekly [39]. Geospatial software (‘@trip pc’ (http://www.a-trip.com) was used to analyse, edit, restructure and filter GNSS tracks.

### Behavioural analysis

Observed behavioural states are described in the ethogram (S2 Table) and were considered mutually exclusive. Instantaneous scan data of flock behaviour (a total of 45 hr of direct observation) were used to calculate time-budgets, that is, the proportion of time spent in each behavioural state. To represent behaviour over an entire hour, 10 min scan data were multiplied by 6. Durations (per hour) of each behaviour were converted to a percentage of the total time.

Focal data of individual behaviour (POV cameras) resulted in a total of 107 (~290 h) video sequences in Experiment 1 and 45 (~123 h) in Experiment 2. Video sequences were independently analysed by three observers. The intra-observer and inter-observer reliability of the video behavioural analysis was determined using the same three randomly sampled videos per week (each in a different patch and with a different animal). Relative reliability was assessed via the intraclass-correlation index and the coefficient of variance (n = 33; 0.67; 0.72).

The full video sequences analysis provided three sets of behavioural data: patterns of grazing, measured as time-budgets of behavioural activities; structure of intake, measured as the number and frequency of events; and diet selected, measured as feeding niches allocated to feeding stations.

Behavioural states were extracted from video sequences through continuous focal sampling using Behavioural Observation Research Interactive Software (BORIS, [40]), a free and open-source event logging software. The full video sequences were analysed using 10 sec intervals to reduce the workload. Whenever the animal changed behavioural state, the video was rewound to pinpoint the exact second to record behaviour continuously. Additionally, whenever the start of a feeding station was logged, the corresponding feeding niche selected by the animal was registered. For each video sequence, the software’s output provided the time-budgets, sequence, total duration, and total frequency of each behaviour.

This data set was further organized hierarchically into grazing, meal, bout, and station patterns, and the structure of each behaviour level was determined, namely the size, duration, and rate (Fig 1). At each behaviour level, activity was separated by intervals (Fig 2). The majority of intervals between meals were longer than 60 minutes, while intervals between bouts were usually longer than 5 minutes but shorter than 60 minutes. We used the hour as the time unit for analysing bout patterns, similar to [41].

In addition to grazing pattern and structure, the video sequences revealed the diet composition, which was estimated from the proportion of feeding niches containing grasses, legumes, and forbs in the total feeding niches used during a meal. Moreover, to estimate diet selection, we used the time spent on each feeding niche and related these durations to the quantitative occurrence of plant functional groups on the patch selected for grazing (c.f. Pasture Measures section). Considering the proportion of feeding time on a feeding niche as equivalent to the proportion of the respective food type in the herbivore’s diet, we assumed a preference for the vegetation type and determined the preference index (Pi; [Eq 3] [42]):

Pi=FNi−Ci/FNi+Ci (3)

where FNi is the proportion of feeding time spent in vegetation of a given food type, and Ci is the proportion of area covered by vegetation of the same food type.

### Statistical analysis

Repeated measures data were analysed using mixed models, with date and time periods as fixed effects, and animals as random effects (SPSS 22.0 software, IBM Corp., Armonk, NY, USA). Variables were tested for normality (Shapiro−Wilk) and homoscedasticity (Levene). When the assumptions were not validated, non-parametric tests were used (Kruskal−Wallis, Spearman’s correlation).

To compare behavioural variables among dates (early spring and late spring 2016, and early spring 2017), weeks were grouped by season (early spring: first 5 weeks; late spring: last 5 weeks). Although a 1-week gap between seasons was used to create two balanced clusters of 5 weeks (used in one-way analysis of variance [ANOVA]), all the tables show all data set (11 weeks). Means were compared using the Bonferroni post-hoc test. Two-way ANOVA was performed each year to evaluate the effects of date or time period on behaviour variables. Means were compared using Tukey’s test. Individual grazing behaviour and group grazing behaviour were compared using Spearman’s rank correlation and t-test (SPSS 22.0 software, IBM Corp., Armonk, NY, USA). The threshold of statistical significance was set at P < 0.05 for all statistical analyses.

### Ethics statement

The experiments were conducted according to the regulations and ethical guidelines of the Portuguese Animal Nutrition and Welfare Commission (DGAV, Lisbon, Portugal) and following the European Directive on the protection of animals used for scientific purposes (2010/63/EU). The study was reviewed and approved by the Scientific Committee of the Institute of Mediterranean Agriculture and Environmental Sciences (ICAAM) under the authorization ICAAM/RCCI 2016.012. The welfare of the sheep was monitored weekly [43]. Field research was carried out at the University of Évora experimental farm with the permission of ZEA (Sociedade Agrícola Unipessoal Lda, Évora, Portugal).

## Results

### Comparison of grazing patterns obtained by POV video recording or direct observation

**Fig 4:**
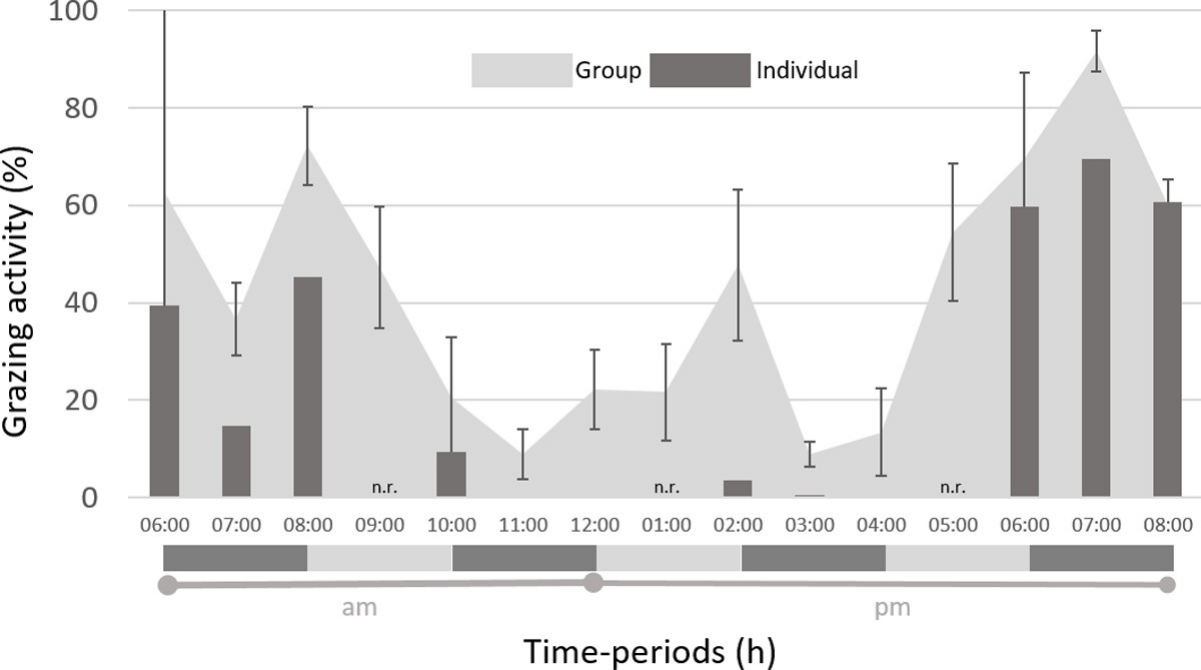
Comparison of daily grazing pattern obtained with POV cameras (individual) or by direct observation (group). Group grazing refers to the proportion of animals grazing in the flock (mean ± standard error of mean [SEM]; n = 15). Individual grazing refers to the proportion of minutes per hour (mean ± SEM; n = 3). n.r. = not recorded.

The grazing behaviour observed for the entire flock was similar (P = 0.126) to that recorded by POV cameras, showing the same diurnal major meals (Fig 4). Moreover, the correlation between grazing activity assessed hourly by individual video recording and direct observation was highly significant (Spearman correlation coefficient = 0.84, n = 24, P < 0.0001). Conversely, for grazing activity outside dawn and dusk major meals, the proportion of grazing was underestimated by POV cameras as a result of differences between animals (P = 0.04). The largest difference measured by the two approaches occurred in the 2–3pm time period when over 40% of ewes were observed grazing, while grazing activity registered by POV cameras was low (3.2%).

### Variation in grazing patterns

**Fig 5:**
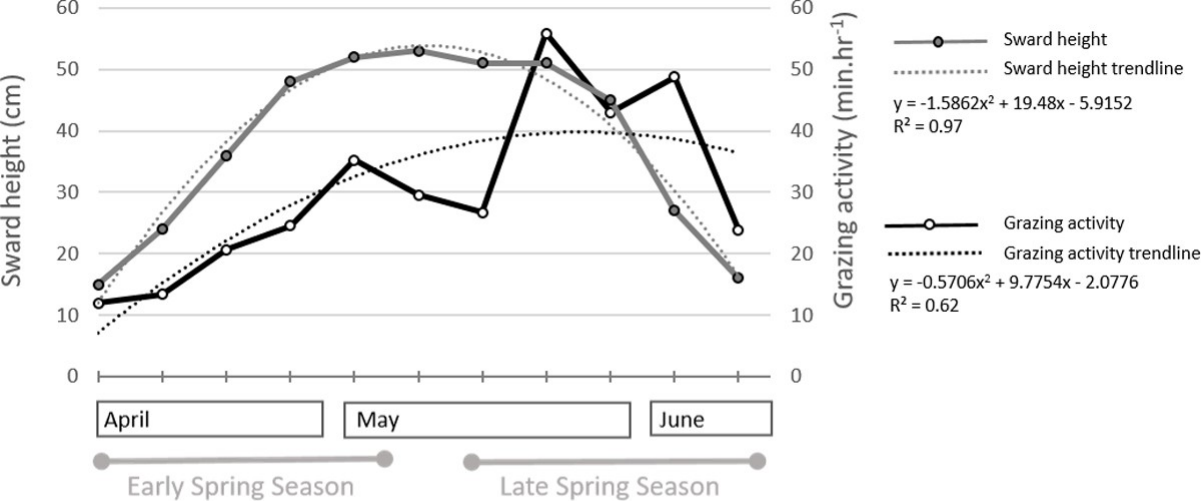
Values and trendlines of sward height (cm) and grazing activity (min.hr^−1^).

Total daily grazing time and sward height increased asymptotically over time (P<0.001 for both trendlines; Fig 5; Table 1). This means that both parameters reached a maximum and then decreased over time. Maximum pasture height occurred about 3 weeks before the maximum daily grazing time.

**Table 1:** Weekly variation in sward characteristics (structure, nutritive value and composition) of patches selected for grazing.

	Forage mass	Sward Height	Bulk density	CP	NDF	PCi	Grass Cover	Legumes Cover	Forbs Cover
Date									
	kg DM.ha^-1^	cm	mg.cm^-3^	%	%		%	%	%
**Early Spring 2016**									
April 4th	1431	15	38.2	15.6	38.8	0.26	9.0	23.0	56.5
April 11th	3520	24	57.9	15.2	47.5	0.22	5.2	77.1	16.7
April 18th	6787	36	75.4	15.8	53.1	0.18	11.6	70.4	15.9
April 26th	3867	48	32.2	12.3	52.8	0.53	20.8	26.0	43.2
May 2nd	4796	52	36.7	11.7	51.6	0.48	15.6	34.4	46.4
May 9th	5262	53	39.6	9.4	58.8	0.63	67.7	8.3	13.0
**Mean**	**4080**	**35**	**48.1**	**14.1** ^**a**^	**48.8** ^**b**^	**0.34**	**17.4** ^**b**^	**41.6** ^**a**^	**33.7**
±SEM	872.2	7.0	8.15	0.88	2.67	0.07	4.79	14.34	8.02
**Late Spring 2016**									
May 16th	4542	51	35.6	9.1	60.1	0.75	72.4	1.0	23.4
May 23rd	7849	51	61.3	10.5	61.3	0.38	39.6	1.6	47.2
May 31st	4796	45	42.4	8.9	58.8	0.62	65.1	2.6	29.7
June 7th	6240	27	91.7	10.8	59.7	0.24	25.0	0.0	67.7
June 13th	7200	16	176.3	10.5	65.7	0.14	77.1	0.0	13.0
**Mean**	**6125**	**38**	**81.5**	**9.9** ^**b**^	**61.1** ^**a**^	**0.43**	**55.8** ^**a**^	**1.0** ^**b**^	**36.2**
±SEM	648.6	7.0	25.64	0.39	1.22	0.11	10.07	0.49	9.64

CP − crude protein; NDF − neutral detergent fibre; PCi − pasture condition index. Seasonal means (± SEM) in bold type. Means within the same variable with different superscripts are significantly different (P < 0.01) among seasons; for the cover variable, differences from 100% correspond to species not identified. Values in rows are sward averages (n = 9). Means for early spring relate to the first 5 weeks only.

In addition to the surface height, other variations in sward occurred across seasons (Table 1). While forage mass (P = 0.09) and bulk density (P = 0.25) did not change significantly, a significant reduction in forage nutritive value (a decrease in protein) (P = 0.002) and an increase in fibre content (P = 0.003) were observed over time. The surface cover of visited patches also changed significantly across seasons, and an increase in grasses (P = 0.003) and a decrease in legume cover (P = 0.004) were observed.

**Fig 6:**
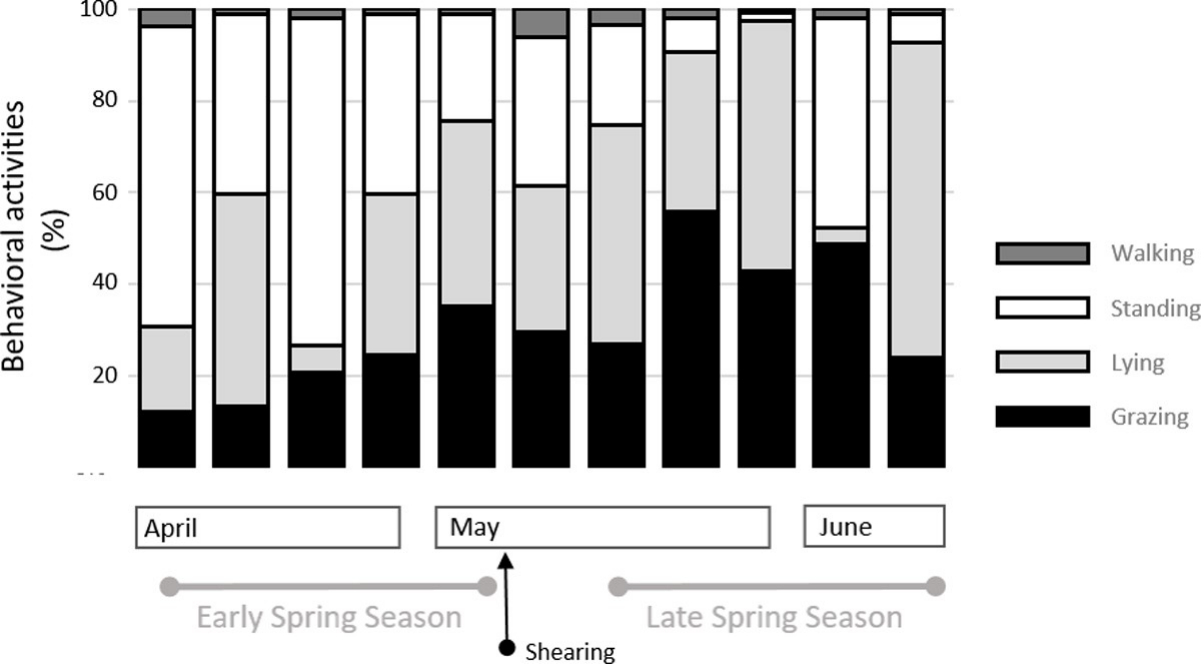
Weekly grazing pattern. Time–budgets of behavioural activities in 3 hr of video recording during major meals. (n = 5).

Increased grazing activity was evident until the ninth week (Fig 5), with the first 5 weeks showing the steepest ascent (trendline y = 5.76x + 3.86; r^2^ = 0.94, P<0.001), pairing the linear increase in sward standing height (trendline y = 9.8x + 5.6; r^2^ = 0.98, P<0.001). The time-budget of grazing activity doubled from early spring (21.2%, ± 9.4) to late spring (40.5%, ± 14.2) (Fig 6). When expressed in min.h^−1^, the total grazing activity ranged from 7.2 min.h^−1^ to 33.6 min.h^−1^ across weeks, with significantly higher meal duration in late spring than in early spring (Table 2). Furthermore, in addition to weekly differences in the meal duration, the daily longer meal occurred at dusk (Table 3).

**Table 2:** Weekly variation in structure of intake (meal and bout patterns).

	Meal pattern			Bout Pattern		
Date	Meal Duration	Meal Size	Inter-bout Interval	Bout duration	Bout Size	Bout rate
	(min.h^-1^)	(n° bouts.h^-1^)	(min.h^-1^)	(min.bout^-1^)	(n° stations.bout^-1^)	(bouts.min^-1^)
**Early Spring 2016**		** **		** **	** **	** **
April 4th	7.2	5	41.6	1.4	13	0.7
April 11th	8.0	1	24.2	6.5	9	0.2
April 18th	12.3	5	44.1	2.7	10	0.4
April 26th	14.6	4	24.3	3.7	11	0.3
May 2nd	21.1	2	14.7	10.8	8	0.1
May 9th	17.6	6	23.1	2.8	4	0.4
**Mean**	**12.6** ^**a**^	**3.3**	**29.8** ^**a**^	**5.0**	**10.0**	**0.3**
±SEM	2.52	0.74	5.63	1.66	0.86	0.11
**Late Spring 2016**		** **		** **	** **	** **
May 16th	16.1	4	15.2	4.6	11	0.2
May 23rd	33.6	5	5.7	6.8	9	0.2
May 31st	22.2	7	1.7	3.2	5	0.3
June 7th	29.2	6	28.6	4.9	18	0.2
June 13th	14.3	2	4.4	6.6	4	0.2
**Mean**	**23.1** ^**b**^	**4.7**	**11.1** ^**b**^	**5.2**	**9.4**	**0.2**
±SEM	3.71	0.86	4.49	0.68	5.59	0.03
**Early Spring 2017 (diurnal) **				** **	** **	** **
April 19th	31.3	21	19.3	1.5	11	0.7
May 3rd	41.3	20	9.7	2.1	15	0.5
May 19th						
**Mean**	**36.3**	**20.5**	**14.5**	**1.8**	**13.0**	**0.6**
±SEM	5.02	0.50	4.84	0.29	2.00	0.09
**Early Spring 2017 (nocturnal) **				** **	** **	** **
April 19th	23.4	17	16.7	1.3	14	0.7
May 3rd	62.2	44	13.1	1.4	10	0.7
May 19th	61.4	61	19.6	1.0	16	1.0
**Mean**	**49.0**	**40.7**	**16.5**	**1.3**	**13.3**	**0.8**
±SEM	12.80	12.67	2.30	0.13	1.76	0.09

Means within the same variable with different superscripts are significantly different (P < 0.01) among seasons. Intake structure analysis was performed within the major meal only.

**Table 3:** Daily variation in structure of intake (meal and bout pattern).

Time period	Meal		Meal		Bout		Bout	
	duration		size		duration		rate	
	(min.h^-1^)	±SEM	(n° bouts)	±SEM	(min.bout^-1^)	±SEM	(bouts.min^-1^)	±SEM
6am-9am	19.5^ab^	5.37	12 ^b^	3	1.7^ab^	26.69	0.7^a^	0.15
10am-1pm	1.6^c^	1.51	2 ^c^	1	0.5^c^	24.14	1.4^c^	1.03
2pm-5pm	0.5^c^	0.40	1 ^c^	1	0.4^c^	11.26	1.6^c^	0.85
6pm-9pm	37.6^a^	1.54	20 ^a^	4	2.3^a^	44.90	0.6^a^	0.13
9pm-6pm	13.1^bc^	6.14	9 ^b^	5	1.4^ab^	22.26	0.4^a^	0.18

Values in rows are averages.hour^−1^ (n = 3). Means (± SEM) within the same variable with different superscripts differ (P < 0.01).

The increase in grazing time was accompanied by a decrease in other behavioural activities. In early spring, animals spent 47.8% (± 20.0) of their daily activities standing. This time-budget was reduced to 16.7% (± 18) in late spring. Conversely, the lying down time-budget was lower in early spring than in late spring (Fig 6).

### Variation in structure of intake

**Fig 7:**
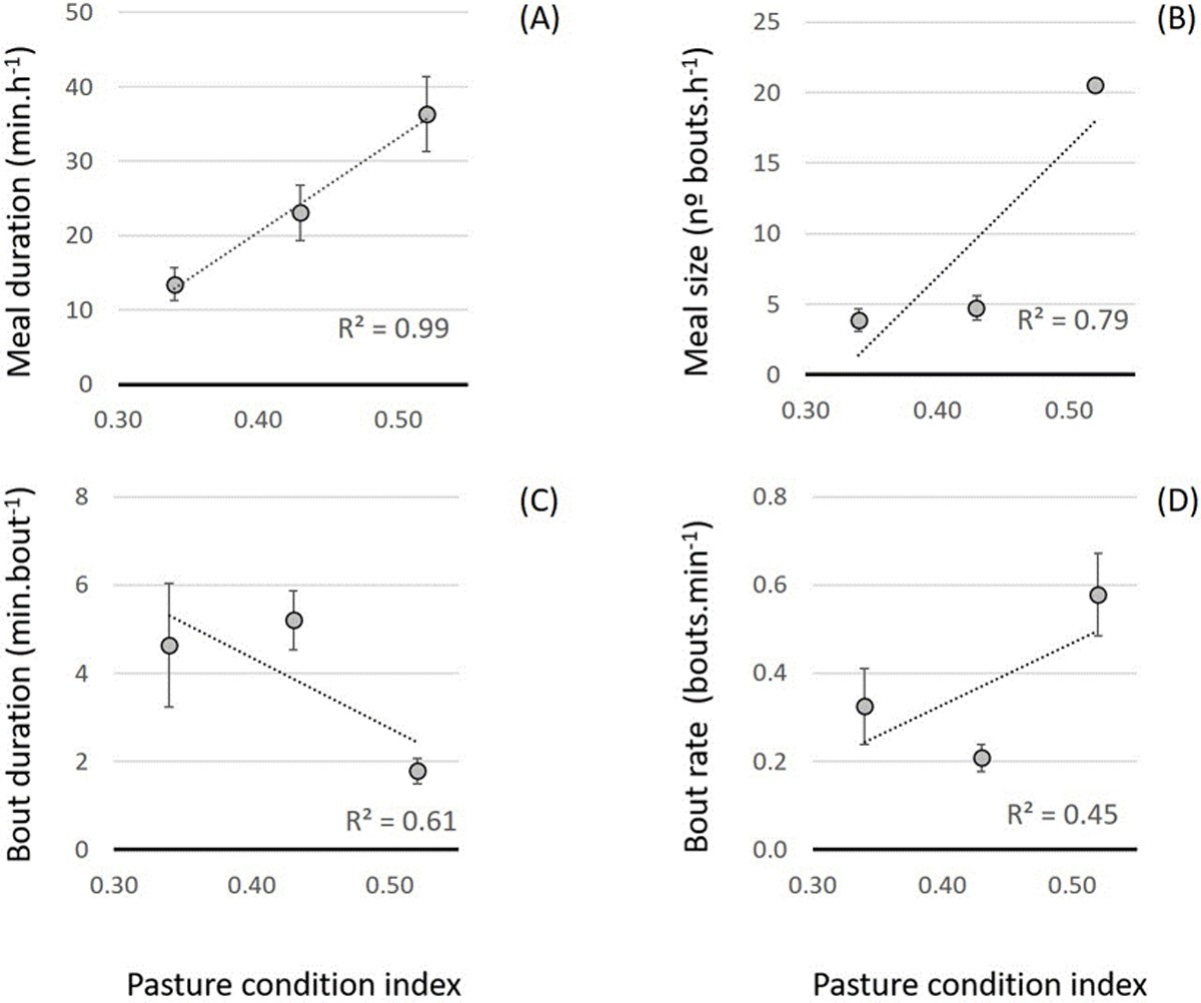
Relationship of structure of intake with plant growth cycle. Vegetative phase, PCi = 0.34 (n = 25; early spring 2016); reproductive phase, PCi = 0.43 (n = 25; late spring 2016); senescent phase, PCi = 0.52 (n = 9; early spring 2017). PCi = pasture condition index. (A) Meal duration; (B) meal size; (C) bout duration; (D) bout rate. The dotted trendline represents the linear estimate of the relationship (P < 0.01).

The weekly structure of feed intake was analysed within the weekly major meal and is presented in Table 2. The relationship between intake structure and plant growth cycle and nutritive value of sward, expressed as PCi, is presented in Fig 7. As sward senesce (PCi = 0.52) meal duration (min.h^−1^) increased (Fig 7A) (r^2^ = 0.99, P < 0.01). Additionally, meal size (number of bouts.hr^−1^) (Fig 7B) increased significantly (r^2^ = 0.79, P < 0.01), 5 times more when compared with the vegetative phase (PCi = 0.34).

Bout duration (min.bout^−1^) and bout rate (bout.min^−1^) had a different relationship to plant growth cycle. Bout duration was highest at the reproductive stage (PCi = 0.43; Fig 7C) paired with a lower bout rate (Fig 7D). Nevertheless, bout rate was highest when PCi was also highest, showing an increasing trendline. Nocturnal grazing showed a different bout pattern from diurnal grazing. Grazing during the night had a higher bout rate and shorter bout durations, as well as a larger meal size and longer duration (Table 2).

Within a single day, the structure of intake differed between meals (Experiment 2; Table 3) in both size and time spent grazing (P < 0.01). The longer meal was in the 6–9pm time period, with significantly more bouts despite grazing and bout duration in that period being similar to those in the 6–9am time period. The bout rate was similar at times of greater grazing activity (6–9am, 6–9pm, and 9–2pm; Table 3).

### Variation in diet composition

**Fig 8:**
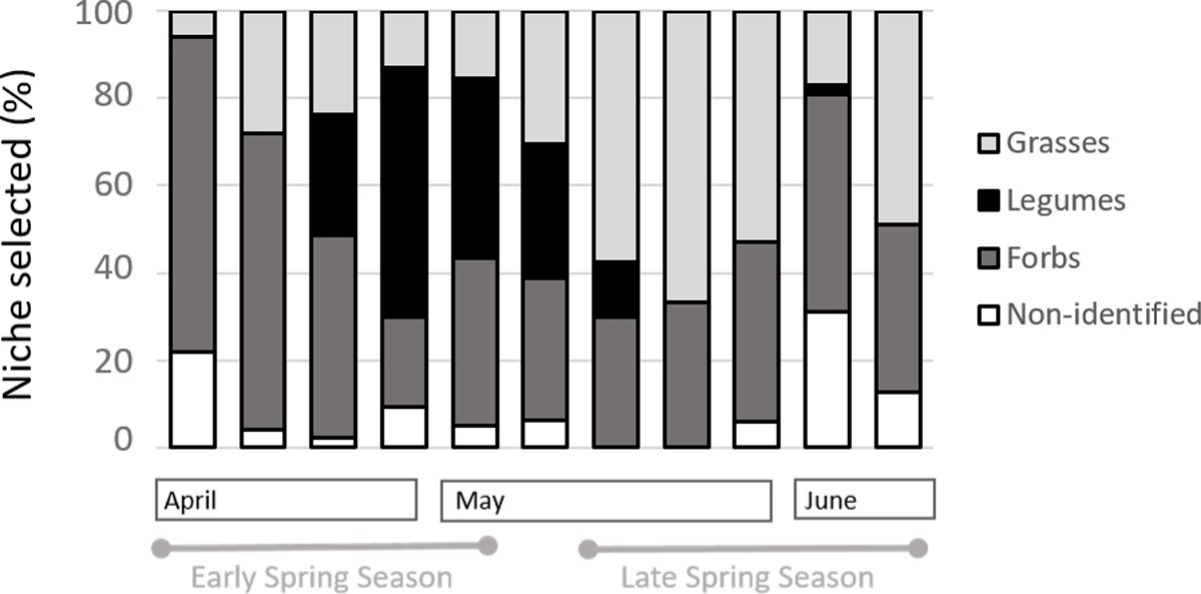
Weekly pattern of diet composition at major meals. Diet estimated from type of feeding niche selected. (n = 5).

**Fig 9:**
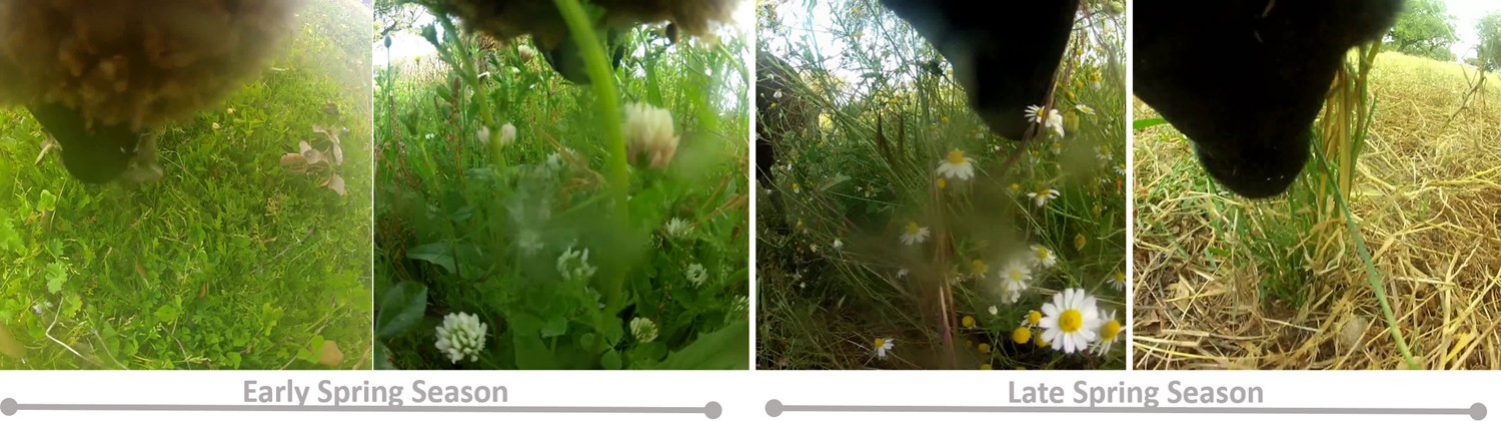
Screenshots of ewes’ dietary choices (feeding niches) in early and late spring. (1) Eating holm oak (*Quercus rotundifolia* [Lam.]) dry leaves; (2) eating legumes (*Trifolium vesiculosum*); (3) eating forbs (*Chamaemelum* spp.); (4) eating grass (*Poa* spp.) (note the differences in sward height and colour).

Diet composition was only evaluated in Experiment 1 (Table 4; Fig 8); examples of feeding niches chosen by ewes are shown in Fig 9. Overall, across the entire season, diet was composed of 42.8% (± 4.7) forbs, 32.6% (± 6.2) grasses and 15.6% (± 6.2) legumes (P < 0.03). The remaining 9% (± 3.7) constituted non-identified species. There was a significant interaction among plant functional groups and seasons (P < 0.01).

**Table 4:** Weekly variation in diet composition, diet selected and station pattern.

	Diet composition			Diet selected			Station pattern	
Date	Niche Grass	Niche Legumes	Niche Forbs	Grasses	Legumes	Forbs	Station Duration	Station Rate
	(%)	(%)	(%)	Pi	Pi	Pi	(s.FS^-1^)	(FS.min^-1^)
**Early Spring 2016**								
April 4th	6.0	0.0	72.0	-0.69	0	0.12	6.0	10.0
April 11th	28.0	0.0	68.0	0.69	-1.00	0.61	5.4	11.1
April 18th	23.7	27.8	46.4	0.34	-0.43	0.49	6.3	9.5
April 26th	12.9	57.1	20.7	-0.24	0.37	-0.23	5.5	10.9
May 2nd	15.3	41.4	38.2	-0.01	0.09	-0.10	8.9	6.7
May 9th	30.6	30.6	32.7	-0.38	0.57	0.43	7.5	8.0
**Mean**	**17.2** ^**a**^	**25.3**	**49.1**	**0.02**	**-0.19**	**0.18**	**6.4**	**9.6**
±SEM	3.91	11.31	9.52	0.18	0.29	0.19	0.65	0.79
**Late Spring 2016**								
May 16th	57.5	12.5	30.0	-0.11	0.85	0.12	6.3	9.5
May 23rd	66.7	0.0	33.3	0.25	-1.00	-0.17	10.6	5.7
May 31st	52.9	0.0	41.2	-0.10	-1.00	0.16	12.0	5.0
June 7th	17.0	2.2	49.6	-0.19	1.00	-0.15	5.5	10.4
June 13th	48.9	0.0	38.5	-0.22	1.00	0.49	9.8	6.1
**Mean**	**48.6** ^**b**^	**2.9**	**38.5**	**-0.08**	**-0.04**	**0.09**	**8.9**	**7.3**
±SEM	8.43	2.43	3.39	0.09	0.50	0.12	1.25	1.16

Means within the same variable with different superscripts are significantly different (P < 0.01) among seasons.

The proportion of grasses in the diet increased (P < 0.01) from 17.2% (± 3.9) to 48.6% (± 8.4) from early to late spring. Although not significantly, legume feeding niches tended to be selected (P < 0.09) more often in early spring (25.3% ± 11.3) than in late spring (2.9% ± 2.4).

Variation in the Pi among weeks was very high for legumes (Table 4), evidencing a strong tendency to ignore legumes at the beginning of the vegetative stage and again at more mature stages. Forbs were preferred to other vegetation types in most weeks. The Pi for forbs and legumes was, on average, higher in early spring than in late spring, when preferences for those groups were close to zero. At this stage, the proportion of feeding time on forbs and legumes equals the proportion of area covered by the same plant functional groups. The Pi for grasses in both seasons was similar and close to zero, meaning that this functional group was never selected or avoided, thus indicating that the intake rate is dependent on the frequency with which the animal encounters grasses.

**Fig 10:**
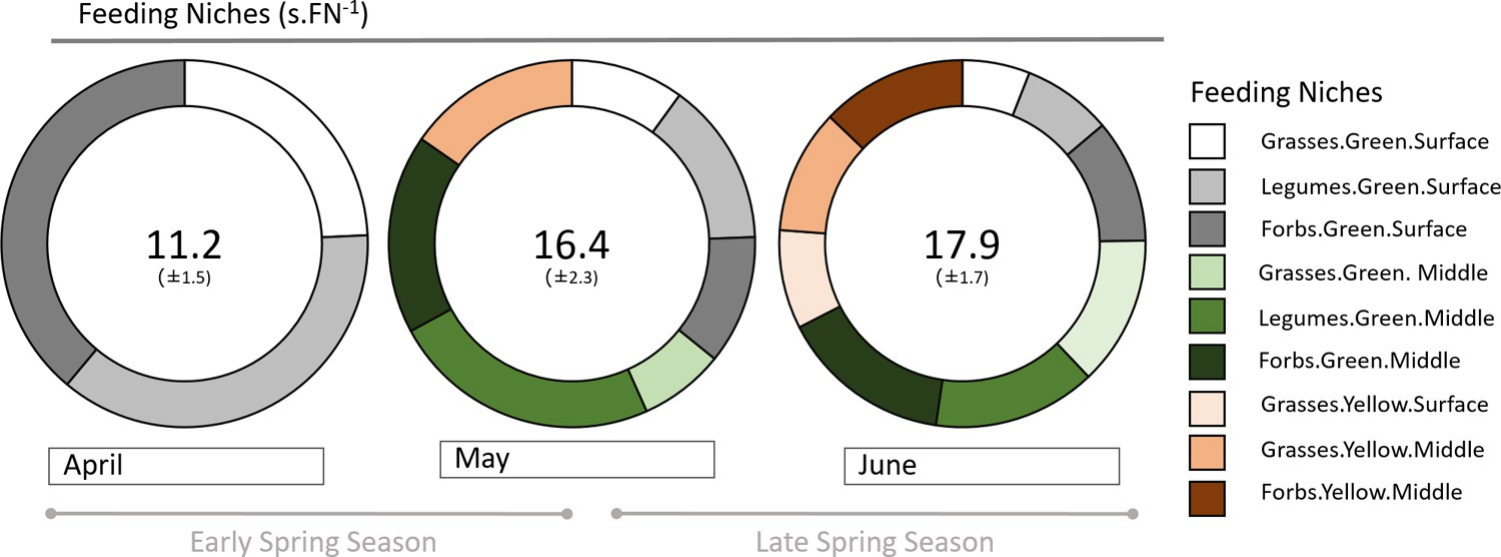
Type of feeding niches selected across plant growth cycle. Station duration within niches selected monthly displayed in the centre of the circle (in seconds: mean ± SEM; April n = 20; May n = 25; June n = 10).

Behavioural variables at station level (station number, duration and rate) were not significantly affected by season (P > 0.05). As the plant growth cycle progressed, sheep grazed more niche types (Fig 10) and preferred green vegetation (70%) to senescent plants.

**Fig 11:**
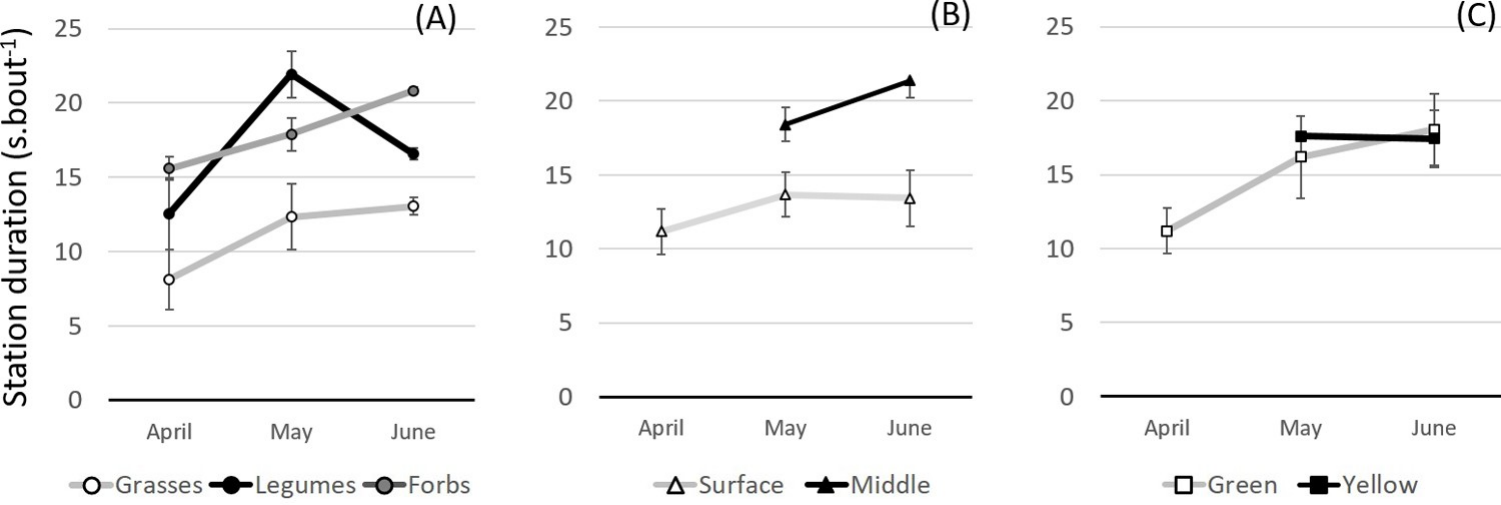
Variation in station duration acording to niche features. (A) Sward functional group; (B) sward vertical strata; (C) plant maturity (mean ± SEM; April n = 20; May n = 25; June n = 10).

Sward attributes, namely plant functional group, plant height, and plant maturity (Fig 11; P > 0.05), influenced station duration differently through the growing season.

In May, sheep spent more time grazing at legume feeding niches than on any other plant functional group (Fig 11A), and they consistently spent less time on grasses. In June, animals spent more time at forbs feeding niches compared with other functional groups. As plants matured and the height of the sward canopy increased, sheep were able to graze in different sward vertical strata and preferred feeding niches at the middle strata (Fig 11B). Moreover, the sward colour changed progressively from green to yellow, and an overlap between station duration in green or yellow feeding niches was observed (Fig 11C).

## Discussion

The primary advantage of using POV cameras to analyse grazing behaviour is the new source of information they provide: the kinetics of intake, that is, the quantifying of changes in grazing behaviour determined by the conditions of vegetation selected at a particular time. In addition to providing a better understanding of the mechanisms through which animal–plant interactions occur, this information can reveal the effects that a change in feeding environment may have on the grazing behaviour. Knowledge of grazing behaviour microstructure is of practical use, as it can provide the basis for predictions that can help in developing strategies for grazing management. For example, studying the animal–plant interactions at the various levels of the feeding behaviour has allowed us to identify transition points in behavioural components.

### Comparing individual and group behaviour

A question underlying the use of POV cameras is: will individual patterns detected with POV cameras be representative of the grazing behaviour of the entire flock? We found that the time-budget of grazing activity showed a significant correlation between the group observations and the observations captured with POV cameras. This indicates that the flock and the animals with POV cameras behave in the same manner, and therefore the POV cameras were suitable for determining grazing behaviour characteristics of the entire group.

Similar results were reported previously [11] after comparing the time devoted to intake by 1 trained ewe or by a group, having found a correlation coefficient of 0.72 when the flock had 214 ewes in a 4.5 ha paddock, and a correlation coefficient of 0.89 with a smaller flock of 25 ewes grazing in a 1.1 ha paddock. Although variation may be found from individual to individual, each individual’s activities appear to be governed by the same dynamics as the population [8, 44]. The matching of individual and group behaviour may be explained by the social behaviour of domestic ruminants, influencing grazing distribution. Both intrinsic species’ behaviour and paddock size could have contributed to the observed results. When compared to cattle, sheep show both more cohesion and behavioural synchronization [45], and grazing animals are known to move more tightly in 1 ha pasture than in larger pastures [46].

Despite the good agreement, it is important to note that the reasons for the underestimation of total grazing time outside the major meal by POV cameras may actually be twofold: not only individuals with POV cameras may have underestimated group behaviour, but also the behaviour of the flock may have been overestimated when using scan sampling. Instantaneous scan sampling is a score and does not provide information on true durations. However, the shorter the scan sample interval gets, the more instantaneous sampling resembles continuous recording [30]. Although it was proved that an instantaneous scan sampling interval of 10 min was able to estimate lying, grazing, and standing behaviour on pasture accurately [47], in our study, average bouts were shorter than 10 min (4.9 min.bout^−1^). A grazing bout identified by direct observation within an interval of 10 min may have led to both overestimation and underestimation of the group behaviour.

Nevertheless, although absolute grazing activities between the individuals and the flock may differ (underestimated in our data and overestimated in the data in [11]), the same meal pattern was observed for individuals and group: two major meals with shorter arbitrary meals between. These short meals are more prone to vary among individuals [26] due to interaction with rumination. A possible explanation is that short meals may start at any moment, regardless of whether the sheep is ruminating or not. This may indicate that short meals are opportunistic, depending on the neighbourhood of the individual resting site [8]. Furthermore, it is worth noting that in addition to the temporal meal distribution, the spatial meal distribution of grazing behaviour between individuals and group were similar, with all the flock grazing in the same site. Sheep grazed close to each other, and the distance between animals did not exceed 1 m (detectable from both the video recordings and direct observation). A similar observation was reported by [31, 48]. The proximity among ewes suggests that each individual ewe was grazing within the same plant community, and it is reasonable to assume that both individuals and group had similar feeding conditions and therefore similar diets. Support for this assumption is provided by a study conducted by [11], which analysed faecal samples using NIR spectroscopy. More recently, studies using faecal DNA metabarcoding to estimate caribou diet composition [49, 50] have shown high correlations with diet estimated by POV cameras. This information supports the assumption that individual animal-borne cameras coupled with GNSS sensors could be used to evaluate the meal spatial pattern of grazing groups.

### Meal pattern in relation to sward characteristics

Patterns are context-dependent, that is, they rely on a background to stand out and are intrinsically linked to the situation in which they are formed. Pattern format provides an analytical framework to apply solutions to complex problems (e.g. it is frequently used in computer science). The principle underlying feeding patterns is the same: by understanding regularities, it is possible to predict what comes next and estimate whether the same pattern will occur when variables are altered [51].

The meal pattern emerges from the relationship between inter-meal intervals and meals. Meals are the main functional unit in which animals organize their feeding behaviour, and analysis of meals produces the most biologically relevant information [23, 52–54] for understanding grazing behaviour. For example, we have found that the time period of major meals changed depending on seasons. From early to late spring, the increase in the inter-meal interval results in a longer interval between the two longer meals of the day. Similar results were reported in the literature [8, 55].

In addition to the meal pattern, the structure of the meal itself changed. We found that meal duration followed changes in sward maturity and increased across seasons (Table 2; Fig 6). The increase in meal duration may result in an increased intake. A linear relationship between minutes of eating and dry matter intake was observed in previous studies [52, 53]. Our results also showed a linear increase in the first 5 weeks of early spring indicating a type I functional response (a linear increase in intake rate with bulk density). The functional response is the consumer’s intake rate as a function of food density [55]. The linear increase indicates that consuming food does not interfere with searching for food [55].

Nevertheless, intake usually decreases as plant maturity progresses, particularly at senescent sward stages [56], meaning that animals can increase meal duration up to the point where the trade-off between quality and quantity starts to prevail. After the first 5 weeks, we also observed a decrease in grazing activity from the ninth week when the sward was senescent (decreasing in height from the sixth week), which was an asymptotic functional response. The type II functional response indicates that processing of food and searching for food are competing and can overlap in time, and are regulating the instantaneous intake rate in response to changes in the size of bites obtained by the grazer [55, 57]. At this transition point, grazing behaviour represents a compromise between chewing forage, which increases passage rate, and biting forage, which increases intake [58]. Therefore, the intake rate (g DM.min^−1^) is related to other rates (i.e. mastication, digestion, passage) that are related to the type of food ingested. For instance, the plant reproductive stage is associated with an increase in the fibre content and altered morphological characteristics, which are accountable for reducing the ease of prehension and the intake rate [26]. To compensate for a reduction in total intake rate, the only effective strategy is to increase grazing time, as shown previously in cows [23]. The same trophic strategy was also found in small ruminants. Nielsen et al. [54] reported that when goats had a 9% reduction in intake rate caused by a high-fibre diet, they reduced their meal frequency by 10% and increased their meal duration by 11%.

In addition to the vegetative and chemical traits of feed resources, other factors can influence the intake rate and meal duration. Meal duration may result from the interaction of different mechanisms; however, in specific feed contexts, some of these mechanisms may be more decisive than others. For example, sward height and forage mass changed from early to late spring (Table 2), and the increase in meal duration can be a consequence of the larger volume bites [48, 53], with more forage in the mouth consequently increasing handling time. In late spring, the sward canopy height and the intake duration decrease; nevertheless, the intake duration remains higher when compared with the beginning of spring (Fig 5). The main mechanism responsible for meal duration in this case is the need to search for and select feeding niches that favour leaf over stems [59], increasing the number of bites. A different functional response (type IV) was observed in heifers grazing monocultures [60]. The increase in meal duration in short sward heights resulted from decreased bite mass instead of longer time needed to encounter and process bites.

Therefore, by using animal-borne POV cameras, we were able to uncover the microstructure of a major grazing meal, that is, within each major meal, we were able to characterize the bout and station patterns in relation to their respective spatial location: the patch and the niche. The bite pattern was explored elsewhere [61] and is outside the scope of the present study. The characterization of a meal in its hierarchical spatiotemporal components provides an understanding of the dynamics of the interactions between plants and animals, resulting in the diet selected and the amount ingested.

### Bout pattern in relation to sward characteristics

The POV cameras coupled with GNSS locations enabled the identification of the patch selected by an animal for grazing. For the bout behaviour that takes place at the patch scale, the sward horizontal availability (i.e. proportion cover of a plant species) influences the rate at which the sheep encounters food items while grazing and, therefore, the time required to find food alternatives [62]. In variable and complex sward, the choice of a feeding patch can be a consequence of a balance between memory and opportunity [7], frequently reflecting the effort to reduce walking energy expenditure rather than a true feed preference. The strategy is consistent with a principle of maximization of energy intake, with selectivity constrained by costs of searching for and discriminating between different forage resources [63, 64].

In fact, sward maturity is frequently confounded with sward height and forage mass, but together with quantity, maturity further changes the plant architecture, the leaf/stem ratio and the chemical composition, increasing the sward complexity. Further complexity results from the different timing of species’ phenological cycle within plant communities, creating additional vegetation heterogeneity and increasing the differences between patches. Two types of spatial memory are used by herbivores to locate food: reference memory and working memory [7]. Reference (long-term) memory is a map-like representation of the foraging environment, and animals can remember locations and food availability for at least 20 days, thus avoiding areas with little or no forage. Working (short-term) memory is used to remember which areas have been visited recently. Using working memory, grazing animals can remember locations that have been grazed for at least 8 hours and associate locations with consequences [7].

In our study, the patches chosen for grazing changed from early to late spring (reported elsewhere, [65]), and this change in animal spatial distribution could be associated with long-term memory and with exploring new areas. Simultaneously, we also observed an increase in the number and rate of bouts. Bouts are located in patch communities, and an increase in the number of bouts may occur as compensation for the loss of forage nutritive value. The consequent longer meals give the animals the opportunity to navigate between patches and graze areas previously ungrazed. Moreover, the search for dispersed preferred plant communities within patches could explain the increase in the bout number. We also found that sheep showed a tendency to reduce bout duration, spending less time in each location (Fig 7). The simultaneous observed increase in the bout rate may be compensation for the reduced duration of each bout. Additionally, we found that the inter-bout interval was significantly shorter, probably due to the reduction in standing time and the increase in walking activity. A similar pattern of more frequent bouts and bouts with short duration was reported by Iason et al. [66] after investigating the ability of grazing sheep to compensate for restricted grazing time. Moreover, the effects of patch encounter rate on patch choice were studied in a laboratory environment using rats [64]. Animals had to choose between available patches that differed in searching/sorting cost. Animals exploited the low-cost patch in almost 100% of encounters. When low-cost patches were rare, the rats did not increase their use of high-cost patches but responded to this increased cost by increasing bouts at both patches, thereby limiting foraging cost and maintaining total intake.

The evidence indicates that the patch selected by sheep is expected to come at a cost which is influenced by the sward horizontal structure, affecting grazing behaviour at the bout level. Consequently, as sward complexity increased, sheep adjusted by having more and shorter bouts.

### Station pattern in relation to sward characteristics

We used niche selection as a proxy for diet composition. The composition of plant communities is largely determined by the coexistence of plant species that define ecological niches. Differentiation of niches is a consequence of plant species’ functional traits. The 12 feeding niches that we defined aimed to capture the horizontal and vertical dimensions of plant availability [62]. The classification also aimed to express features that constitute cues for a grazing animal (e.g. visual discrimination, flavour, ease of handling).

Despite its importance for patch selection, sward height was not used by ewes as a definer category for diet selection [67]. Instead, ewes could categorize plants at the species level and used this mental categorization when selecting their diet in the short term. Sheep relied on relevant criteria to create a hierarchical representation of food categories, namely nested levels of perceptual information [67], thus reducing the decision demand and increasing their foraging efficiency when selecting from many different plant items. The species’ plant traits are mostly linked to the botanical family, which will define plants’ physiological and chemical traits. Grasses, legumes, and forbs, differ functionally in vegetation communities. Plants from these functional types differ in their colour and architecture (e.g. stem and leaf form, branching), which may be linked to potential visual cues. They also differ in their chemical characteristics, linked to potential olfactory, gustatory, and textural cues, and simultaneously linked to nutritive value and food reward value [68].

Therefore, we used plant functional groups to characterize niches, and their presence within patches created a mosaic at the sward horizontal dimension. This dimension was combined with variations in the sward vertical dimension. The main driver of variations in this dimension is the plant growth cycle, which generates both distinct vertical forage mass layers (e.g. different leaf/stem ratio) and distinct vertical gradients of chemical constituents [69].

Our results indicated that the sheep’s diet composition varied greatly according to seasonal variations in sward vegetation availability. This was also reported in a study on complex natural grassland [70]. As the sward’s structural complexity increases with seasonal variation, palatability differences between plants and plant fractions within a feeding niche will be more pronounced [48]. Differences in palatability will lead to selective grazing. In other words, the animal will graze the species and parts of plants that it prefers up to the point where the searching cost prevails. In our study, the major change in diet composition was related to the preference for legumes, which was higher at intermediate than at short or tall sward heights. As legumes progressed from vegetative to reproductive stages, a decrease in relative preference was also observed by [18, 71, 72]. Furthermore, it has been reported that sheep prefer swards with intermediate levels of clover, avoiding those with low or high clover levels [73]. Low preference for legumes at the beginning of the early spring season may be linked to low palatability related to a low water-soluble carbohydrate and/or a high nitrate concentration in plants, the latter known by its bitter taste [74]. Low preference for legumes in late spring could, in turn, be a consequence of handling time (in searching and mastication) due to an increase in legumes’ branching and stem/leaf ratio, which increase the discrimination difficulties, thus influencing components of the functional response such as bite depth and bite volume [73].

In this study, it was found that forbs seemed to be preferred to legumes and grasses. Although there are large differences between individual forb species, these non-leguminous dicotyledons generally provide high-quality herbage in mixed herbaceous pastures during periods of low forage mass availability [75–77]. This is possible since forb species acquire and use nutrients at different times and from different resource sites relative to co-occurring grasses and legumes in complex pasture communities [75]. When actively growing, forbs are more digestible and are usually higher in crude protein and phosphorus and lower in fibre than grasses [76]. Because of their low fibre concentrations, forb leaves break down quickly in the rumen, which enables a higher intake than grasses [76]. This chemical and functional role of forbs in the diet could justify their relatively high (about 44%) presence in the diet across spring. As such, while legumes almost disappeared from diets in late spring, forbs remained present, as confirmed by their preference indices, which were always positive. The wide range of maturation of different forb species, providing a continuous feed resource, may also explain our results.

In addition to being influenced by differences in the preferences for functional plant groups, the diet composition across seasons was related to changes in the sward vertical dimension. As sward matured, it was noticed that sheep preferred the middle sward layer for cropping forage, reflecting a preference for greener and prostrated vegetation, such as legumes and forbs [75, 77]. Eating in the middle stratum can also correspond to an increase in searching for leaf mass rather than reproductive plant parts. As plants mature, the proportion of stems increases and interferes with the grasping of leaves by animals, mainly reducing bite mass [59, 73, 78]. Regardless of the reason, the increase in selective grazing was supported by the observed increase in the number of feeding niches used by sheep as pasture matured. In these circumstances, sheep increased the station duration and reduced the station rate, which means that in late spring, ewes spent more time at each feeding niche, probably due to an increase in handling time. More time is probably needed to search among neighbouring plants and select plants, as well as to discriminate among plant parts. Another concerning factor is an increase in plants’ NDF content, which requires more chewing time while in the same feeding station [79].

Nevertheless, although handling time may increase in late spring, preferences decrease. The observed decrease in the Pi of legumes and forbs indicates that animals were more limited in their choices. Similar to our results, another study [80] reported a decrease in overall plant selectivity as summer progresses. The reduction in selectivity and feeding preferences could be explained by the changes in sward surface height occurring in late spring in our annual Mediterranean sward conditions. The loss of forage mass is due to leaf and stem senescence, and they become pale green and finally yellow and fall onto the soil surface, thus increasing the litter layer. As the sward became shorter and less dense, a reduction in intake rate was expected due to lower bite mass [78], as sward height is a key factor to define bite mass because of its direct relation with bite depth and volume [73, 78, 79]. Ultimately, the composition of diet ingested will result from the balance between preferences and handling cost, with the cost–benefit ratio influencing food choices [55, 57].

### Advantages and future uses of POV cameras

The use of POV cameras has several practical advantages. First, they are easy to install and use with sheep. The ewes’ behaviour displayed while carrying sensors, observed both directly and in video sequences, prove that since the first day of their use, ewes did not show any signs of discomfort or try to get rid of the sensors (e.g. shaking heads or brushing against surfaces). Second, the possibility of going backwards and forwards in slow motion to review the images enables a more informed decision about selected feeding niches. Although lip movements were not visible, when an animal made a head push to rip the herbage, part of the bite mass could stick out of the mouth, facilitating plant identification, especially when plants are in the reproductive stage. Even images captured by an infrared camera enable discrimination of feeding niches through plant traits (except for colour).

However, the time required to analyse video sequences is a drawback. In most cases, the editing time of behavioural states, such as walking, standing, and lying down, is 12 times shorter than the ‘real’ video sequence length. For example, a 60 min ‘lying down’ sequence can take about 5 min to analyse. However, video sequences of grazing states require considerably more time (about three times the ‘real’ video sequence length). Identification of feeding niches and analysis of grazing structure often require slow motion to review images, which increases editing time.

Another advantage of POV cameras is their capacity to capture information where an observer would have difficulty assigning a bite code. For example, swards with high, dense forage canopies can be uncovered by POV cameras even when animals graze in the middle vertical vegetation strata. In our experimental site, the native pasture height peaked at 53 cm, with a bulk density of 176 mg DM cm^−3^. With an average height of 70 cm–80 cm, sheep are ‘immersed’ in the forage, hampering the direct observation of their mouths required for the coding grid methodology. Although our feeding niches approach was unable to differentiate among bite categories, it could discriminate feeding scenarios and recognize the importance of plant tri-dimensional traits in the diet selection.

The information disclosed by POV cameras coupled with GNSS could have two main future uses. One is related to the selection of more resilient and efficient grazing animals. Among the factors influencing feed efficiency, feeding behaviour can account for 35% of the variation in DM intake [81]. Individual differences in intake behaviour are well known. Dado and Allen [82] reported variations among several intake behaviour components, with coefficients of variation across cows ranging from 5% to 41%. The individual variations observed among animals grazing the same feeding niches can be useful to study the plasticity of individuals to feeding conditions. This could prove useful in breeding programmes, as more resilient animals can be more efficient in rangeland conditions. Some animals are able to maintain a high intake rate in a less favourable feeding environment and maintain their live weight, as noted in residual feed intake studies [83, 84]. In other words, despite changing the intake rate, animals can maintain the same intake by increasing bout frequency [54].

Another future application of POV cameras is for gathering contextual information, linking pasture and animals, for the design of new pastures. The sward structure is a central determinant of primary and secondary productivity of grazed ecosystems. As such, approaching the sward structure within a functional framework, which provides an understanding of the most important aspects of plant growth and defoliation under grazing, is critical. For example, overgrazing can occur and will affect the sward structure in the long term, whenever animals graze their favourite patches repeatedly, having a greater impact on their preferred plants. Understanding the dynamics of grazing patterns in specific feeding contexts would be useful to design pastures based on knowledge of plant ecophysiology and the microstructure of grazing behaviour. Pastures could be designed with the objective of modifying food intake and the preference of herbivores, enabling innovative grazing systems and management strategies, consequently contributing to improving the integrity of natural landscapes and ecosystems [85, 86].

## Conclusion

Simultaneous assessment of vegetation features and the corresponding animal behavioural response is essential to assess the dynamics of the resource–consumer interaction. A major strength of this study is that it provides a framework to uncover the meal microstructure of animals grazing in complex swards. Another strength of the present study is it demonstrates that POV cameras can capture the link between grazing behaviour components and sward features.

Overall, the results demonstrate that sheep were able to adapt grazing behaviour to sward changes. Sheep compensated for the decrease in sward quantity and nutritive value by increasing the size and duration at each behavioural scale (i.e. meal, bout and station) while increasing the bout rate and reducing the station rate.

The diet composition changed with sward seasonal changes, and ewes showed the strongest preference for niches that contained forbs, in the middle strata, with green plant material to those that contained grasses, in the surface strata, with mature plant material. Selectivity increased with sward height, which is supported by the increase in niches used for grazing and, simultaneously, the increase in station duration. The latter could indicate a slower intake rate when feeding in mature complex swards. Therefore, our results support the hypothesis tested, providing evidence of the validity of the use of POV cameras as a tool to study the microstructure of intake and the diet composition in free range grazing.

It is reasonable to assume that the microstructure captured by POV cameras in our study was representative of grazing behaviour in this complex sward. An accurate estimation of diet composition enables us to better predict grazing impacts upon a given vegetation community, and POV cameras could be a useful tool for ecosystems sustainability assessment. Furthermore, POV cameras enabled the identification of different transition points in both the frequency and duration of the components of the grazing behaviour. These transition points occurred at the different behavioural scales (oral, station, bout, and meal) and should be further investigated, as they may provide relevant information for future innovations in tailored pasture design (e.g. managing patches to create a functional mosaic of feed resources to achieve animal distribution that positively impacts the pasture ecosystem). Individual differences in grazing behaviour can be better explored using POV cameras and presents another opportunity for future research. Exploring how animals are coping with the environment could contribute to a better understanding of individual feed efficiency, as well as behavioural plasticity within populations. Therefore, POV cameras could be a useful tool to uncover the relationships between an animal resilience and its long-term efficiency that could be used in breeding programmes.

A limitation of this study is that temporal patterns were derived from the grazing relations in just one wood pasture, and therefore the findings may not be generalizable to other swards with a different botanical composition and tree cover. Although the diversity of abiotic and biotic factors can influence the botanical composition of swards, forage mass, plant height, and stage of maturity, we believe that the framework proposed is useful. Relating the microstructure of intake to pasture attributes expressed as PCi (used as a benchmark), in any context, generates relevant information on the dynamics of the plant–animal interaction. Further studies involving a larger number of eating scenarios are needed.

## Supporting information

S1 Table Glossary. For the purposes of this paper, the following terms and definitions apply.
(DOCX)

S2 Table Ethogram of observed behavioural states captured by direct observation or by video recording. (DOCX)
