# Supplementary material for: Characterization of grazing behaviour microstructure using point-of-view cameras
Source: PLoS One. 2022 Mar 18;17(3):e0265037. doi: 10.1371/journal.pone.0265037 (PMC8932577; doi:10.1371/journal.pone.0265037)
Supplement: S1 Table — For the purposes of this paper, the following terms and definitions apply. (DOCX) [file pone.0265037.s001.docx]

**S1 Table. Glossary. For the purposes of this paper, the following terms and definitions apply.**

| Term | Definition |
| --- | --- |
| Bite | The act or action of cutting, piercing or wounding with the teeth (Gibb, 1998). |
| Bite pattern | Defined from the bite rate and bite volume. Daily intake is calculated as the product of total grazing time, intake per bite (bite volume) and bite rate. Plant flavor and texture affects bite pattern. |
| Bout | The length of time that an animal eats without stopping. A bout is a unit of the hierarchical structure of grazing behaviour, characterize by a frequency and a duration. Bout last from 1- 30 minutes. (Corresponds to grazing event in Gibb, 1998). |
| Bout pattern | Defined by the spatial and a temporal dimensions of the bout. Bout pattern results from the distribution of stations and inter-station intervals. |
| Chew | The activity of crushing, bruising and grinding food with the molars. The food may be that just severed or a regurgitated bolus. (Corresponds to mastication in Gibb, 1998). |
| Grazing | Activity that includes eating and also short periods when the animal is not actively eating but is engaged in activities directly associated with eating, such as searching or moving from one patch to another (Gibb, 1998). |
| Grazing pattern | Grazing patterns have a spatial and a temporal dimension and results from the distribution between meals and inter-meal intervals. Grazing pattern may arise from decision-making processes in response to external stimuli, such as environmental conditions or landscape features. The animal’s decisions for grazing defines a foraging strategy and can be observed as grazing patterns. |
| Inter-bout interval | Breaks in eating activity of more than 5 minutes. These breaks can be considered as separations between bouts. |
| Inter-meal interval | Breaks in eating activity of more than 1 hour. Other activities occur during the inter-meal interval such as walking, standing or lying down with or without rumination. |
| Inter-station interval | Breaks in grazing activity of less than 5 minutes. These breaks can be considered as separations between stations, normally resulting from steps taken between niches. |
| Meal | The activity of eating. Eating is the acquisition of herbage into the mouth, its chewing and subsequent swallowing. A meal is a unit of the hierarchical structure of grazing behaviour, characterize by a frequency and a duration. Meals last from 1- 4 hours. |
| Meal pattern | Defined by the spatial and temporal dimensions of the meal. Meal pattern results from the distribution of bouts and inter-bout intervals. |
| Microstructure of intake | The study of kinetics of food intake during feeding episodes obtained by a parallel recording of behavioural responses to feeding conditions. The method allows to disaggregate feeding episodes at different hierarchical scales (meals, bouts, stations and bites) and to quantify and characterize each of the scale |
| Term | Definition |
|  | levels according to varying feed conditions. At each scale, descriptions include frequency, duration, rate, and pattern. |
| Niche | Locations corresponding to vegetation entities. The niche of a grazing animal within a sward is the location where interactions between the animal and its surrounding environment occur through the mouth. The niche depends on how the animal reacts to the distribution and abundance of plants, and in turn how it alters them. |
| Pasture | A type of grazing management unit enclosed and separated from other areas by fencing or other barriers and devoted to the production of forage for harvest primarily by grazing (Allen et al., 2011) |
| Patch | Location inside a site with similar vegetation characteristics, related with plant communities, defined as a relatively homogeneous area that differs from its surroundings mainly due to structural internal variables such as the number of plants, number of plant species or height of plants. |
| Site | Location inside a pasture, defined by physiographic, edaphic and microclimatic factors such as orography, soil type, humidity, temperature and wind characteristics adding variability to vegetation community types. |
| Station | A unit in the hierarchical structure of grazing behaviour, within a grazing bout, that occurs whenever an animal stops his walking movement to bite plants in a feeding niche. Stations are actions and not locations. When in station, an animal can reach available plants without moving its front feet. A station terminates when the animal starts walking. A station is characterize by a frequency and a duration and last from 5-100 seconds. |
| Station pattern | Defined by the spatial and temporal dimensions of the station. Station pattern results from the distribution of bites and inter-bite intervals. |

Allen VG, Batello C, Berretta EJ, Hodgson J, Kothmann M, Li X, McIvor J, Milne J, Morris C, Peeters A, Sanderson M. An international terminology for grazing lands and grazing animals. Grass and forage science. 2011 Mar 1;66(1):2. [doi.org/10.1111/j.1365-2494.2010.00780.x](https://doi.org/10.1111/j.1365-2494.2010.00780.x)

Gibb MJ. Animal grazing/intake terminology and definitions. Pasture Ecology and Animal Intake. Teagasc, Grange Research Centre, Dunsany, Ireland. 1998:21-37. doi.org/10.13140/2.1.3335.8726
