# Supplementary material for: Characterization of grazing behaviour microstructure using point-of-view cameras
Source: PLoS One. 2022 Mar 18;17(3):e0265037. doi: 10.1371/journal.pone.0265037 (PMC8932577; doi:10.1371/journal.pone.0265037)
Supplement: S2 Table — (DOCX) [file pone.0265037.s002.docx]

**S2 Table. Ethogram of observed behavioural states captured by direct observation or by video recording.**

| Behaviour | Description of behaviour in direct observation | Description of behaviour in video sequences |
| --- | --- | --- |
| Grazing | Animal is biting the grass, chewing or swallowing it. Animal stands or moves with the head at the level of herbage. Can raise head for less than 10 s while chewing or swallowing herbage. Can walk between stations less than 5 minutes. | Image show the chin near the ground or immersed in herbage. Chewing sounds are heard. Stretching with the head when animal rip the forage. Muscle movements are seen in the ventral surface of the mandible when biting. Can walk between stations less than 5 minutes. When taking steps between stations, the ground and pasture are seen. |
| Walking | Moving more than five minutes in one direction, head erected and orientated ahead, without displaying rumination and without running. | Image focus distance is more than 0.5 m and images moves along the head of the animal. The horizon line and other animals nearby may be visible. |
| Standing | Animal is not moving, staying in station, with all four feet on the ground. Animal can ruminate or have a passive or vigilant posture. | The horizon line is visible. Animal may ruminate (chin with lateral movements and audible sound), or being idle (image stays the same more than 30 s, remaining silent) or being vigilant (image focus change frequently from subject to subject). |
| Lying down | Lying on the sternum or lateral recumbency with all limbs under the body or one extended. May ruminate or being vigilant. | Vegetation is seen at the ground level. Animal may have the head up while ruminating or doing nothing. Animal can have the head on the ground either bent or not. |
| Other active behaviours | Grooming, scratching, stretching, drinking, defecating, urinating, head butting, head lateral displacement, sniffing/licking structures, etc, either while standing or lying | Repetitive motions of head movements such a shaking (when grooming), others behaviours are visible (vision of water when drinking, head bunting, licking, etc. Urination and defecation are not visible. |
